# Prevalence of neonatal hypothermia and its associated factors in East Africa: a systematic review and meta-analysis

**DOI:** 10.1186/s12887-020-02024-w

**Published:** 2020-04-03

**Authors:** Biruk Beletew, Ayelign Mengesha, Mesfin Wudu, Melese Abate

**Affiliations:** 1 Department of Nursing, College of Health Sciences, Woldia University, P.O.Box 400, Woldia, Ethiopia; 2 Department of Medical Laboratory Science, College of Health Sciences, Woldia University, P.O.Box 400, Woldia, Ethiopia

**Keywords:** Neonates, Hypothermia, Determinants, Eastern Africa, Meta-analysis

## Abstract

**Background:**

Neonatal hypothermia is a global health problem and a major factor for neonatal morbidity and mortality, especially in low and middle-income countries. Therefore, this systematic review and meta-analysis aimed to assess the prevalence of neonatal hypothermia and its associated factors in Eastern Africa.

**Methods:**

We used the Preferred Reporting Items for Systematic Review and Meta-Analysis (PRISMA) guidelines to search electronic databases (PubMed, Cochrane Library and Google Scholar; date of last search: 15 October 2019) for studies reporting the prevalence and associated factors of neonatal hypothermia. The data was extracted in the excel sheet considering prevalence, and categories of associated factors reported. A weighted inverse variance random-effects model was used to estimate the magnitude and the effect size of factors associated with hypothermia. The subgroup analysis was done by country, year of publication, and study design.

**Results:**

A total of 12 potential studies with 20,911 participants were used for the analysis. The pooled prevalence of neonatal hypothermia in East Africa was found to be 57.2% (95%CI; 39.5–75.0). Delay in initiation of breastfeeding (adjusted Odds Ratio(aOR) = 2.83; 95% CI: 1.40–4.26), having neonatal health problem (aOR = 2.68; 95% CI: 1.21–4.15), being low birth weight (aOR =2.16; 95%CI: 1.03–3.29), being preterm(aOR = 4.01; 95%CI: 3.02–5.00), and nighttime delivery (aOR = 4.01; 95% CI:3.02–5.00) were identified associated factors which significantly raises the risk of neonatal hypothermia.

**Conclusions:**

The prevalence of neonatal hypothermia in Eastern Africa remains high. Delay in initiation of breastfeeding, having a neonatal health problem, being low birth weight, preterm, and nighttime delivery were identified associated factors that significantly raises the risk of neonatal hypothermia.

## Background

According to the World Health Organization (WHO), neonatal hypothermia is defined as a core body temperature < 36.5 °C or a skin temperature < 36 °C and is categorized into three levels of severity: mild or cold stress (core 36.0 to 36.4 °C), moderate (core 32.0 to 35.9 °C) and severe (core < 32 °C) [1, 2]. Newborn hypothermia is a global health problem with higher rates in countries with low resource settings [3] and can subsequently lead to diverse neonatal health consequences. In hospital and home settings, prevalence varies from 32 to 85% [4] and from 11 to 92% respectively, and this situation is more challenging in tropical environments [5].

Neonatal hypothermia was associated with a five-fold higher in mortality during the first 5 days of life [6]. Previous studies had revealed that every one degree centigrade decrement of neonate’s body temperature increases the mortality risk by 80 % [3, 6, 7]. From few Sub-Saharan African countries, the hypothermia associated mortality rate was reported to be, 8.1%(community-based study) to 94.9%(hospital-based study) in Guinea Bissau [3], West Africa, 8.1% [7], and in Sothern Nepal, 2.9 to 94.9% [6]. Hence, increasingly, neonatal hypothermia is documented as a contributor to newborn survival [8, 9]. Several conditions of immature thermal regulation, such as low birth weight (LBW), prematurity, intrauterine growth restriction, and asphyxia (with heat loss due to lack of oxygenation, where attempted during reanimation efforts) during birth are significantly associated with abnormal low body temperature [10, 11]. Inadequate intra-natal and postnatal care is another factor which contributes for the onset of neonatal hypothermia [12]. Newborn bathing within the first day after birth, poor socioeconomic status, pitiable kangaroo mother care practices, initiation of breastfeeding after 1 hour, massage of neonates with oil and insufficient health worker’s knowledge on thermal care were determinant factors for neonatal hypothermia [13, 14]**.**

In developed countries, neonatal hypothermia accounted for 28% of the world’s burden [15]. Annual neonatal mortality rates (NMRs) vary widely across the world, but West Central Africa and South Asia accounted for the highest NMRs in 2017 [16]. More than 98% of yearly neonatal mortality occurred in developing countries [17]. Identifying the determinants of neonatal hypothermia have a greater input to attain sustainable development goal (SDG-3) of ensuring healthy lives and promote well-being for all at all age.

Interventions addressing hypothermia management and resuscitation might have a substantial impact on neonatal mortality prevention. Indeed, approaches that can prevent and treat neonates with hypothermia are vital to hasten the advancement of newborn survival. In East Africa, previous studies reported the prevalence of neonatal hypothermia which was ranged from 1.3% [18] to 79% [14]. This indicates, there is inconsistency reports of the prevalence of neonatal hypothermia, and prevalence the estimates of its determinants across different geographical settings. Moreover, there is no regionally denoted pooled data in East Africa which uses as a baseline in designing strategies for prevention and control of neonatal hypothermia. Therefore, this systematic review and meta-analysis were aimed to estimate the pooled prevalence of neonatal hypothermia and associated risk factors in the East African context.

### Review question

The review questions of this systematic review and meta-analysis were:

- What is the prevalence of neonatal hypothermia in East Africa?
- What are the determinates of neonatal hypothermia in East Africa?

## Methods

### PROSEPERO registration

The protocol of this systematic review and meta-analysis was registered at the Prospero with a registration number of (PROSPERO 2019: CRD42019131654) that is available from https://www.crd.york.ac.uk/prospero/display_record.php?ID=CRD42019131654.

### Search strategy

This review identified studies that provide data on the prevalence and/or risk factors for neonatal hypothermia with the context of Eastern Africa. In the searching engine, PubMed, Google Scholar, Cochrane library, research gate, and institutional repositories were retrieved. The search included keywords that are the combinations of population, condition/outcome, context, and exposures. A snowball searching for the references of relevant papers for linked articles was also performed. Those search terms or phrases including were: “newborn”, “neonate”, “infant”, “hypothermia”, “low body temperature”, “thermoregulation”, body temperature regulation, and Eastern Africa. Using those key terms, the following search map was applied: (prevalence OR magnitude) AND (causes OR determinants OR associated factors OR predictors) AND (newborn [MeSH Terms] OR neonate OR infant OR child OR children) AND (hypothermia [MeSH Terms] OR low body temperature OR thermoregulation OR body temperature regulation) AND (Eastern Africa) OR developing country on PubMed database (Table S1). Thus, the PubMed search combines #1 AND #2 AND #3 AND #4 AND #5 (Table S1). These search terms were further paired with the names of each East African countries. On both Cochran Library and Google scholar, a build-in text search was used on the advanced search section of the sources. Thus, the key searching terms were considering Eastern Africa countries that compose of Ethiopia, Djibouti, Somalia, Eritrea, Sudan, Kenya, and Uganda. The searching date was January 2000 to December 2019.

### Study selection and screening

The retrieved studies were exported to Endnote version 8 reference managers to remove duplicate studies. Two investigators (BBA and AMK) independently screened the selected studies using article’s title and abstracts before retrieval of full-text papers. We used pre-specified inclusion criteria to further screen the full-text articles. Disagreements were discussed during a consensus meeting with other reviewers (MWK and MAR) for the final selection of studies to be included in the systematic review and meta-analysis.

### Inclusion and exclusion criteria

New-born babies (any gestation) born in hospital settings having core body temperature < 36.5 C within 28 days of birth were included. All observational studies (cross-sectional, case-control, and cohort) were included. Those studies had reported the prevalence and/or at least one associated factor for neonatal hypothermia and published in the English language from January 2000 to December 2019 were considered. Studies which didn’t report the prevalence and /or odds ratio in their result were excluded. Studies conducted on marginalized groups/populations like neonates from mothers with any medical diseases, chronic diseases, or street mothers were excluded. Citations without abstract and/or full-text, anonymous reports, editorials, and qualitative studies were excluded from the analysis. The Prevalence of hypothermia was considered as the proportion of neonates who have core body temperature below 36.5-degree centigrade among the general live birth of neonates within a specific population and multiply by 100 to be prevalence report.

### Quality assessment

The authors appraised the quality of the studies by using the Joanna Briggs Institute (JBI) quality appraisal checklist [19]. There was a team of four reviewers and the papers were split amongst the team. Each paper was then assessed by two reviewers and any disagreements were discussed with the third and the fourth reviewers. Studies were considered as low risk or good quality when it scored 4 and above for all designs (cross-sectional, case-control, and cohort) [19], whereas the studies scored3 and below were considered as high risk or poor quality (Table S2). Furthermore, we thoroughly extract adjusted confounders and main findings from all included studies (Table S3).

### Data extraction

The authors developed a data extraction form on the excel sheet and the following data were extracted for eligible studies: year of publication, country, setting, study design, the definition of hypothermia, adjusted co-founders, the odd ratio of factors, and main findings. The data extraction sheet was piloted using 4 papers randomly, and it was adjusted after piloted the template. Two of the authors extracted the data using the extraction form in collaboration. The third and fourth authors checked the correctness of the data independently. Any disagreements between reviewers were resolved through discussions with third and fourth reviewers when required. The mistyping of data was resolved through crosschecking with the included papers.

### Synthesis of results

The authors transformed the data to STATA 14 for analysis after it was extracted in an excel sheet considering prevalence, and categories of associated factors reported. We pooled the overall prevalence estimates of neonatal hypothermia by a random effect meta-analysis model. We examined the heterogeneity of effect size using the Q statistic and the I^2^ statistics. In this study, the I^2^statistic value of zero indicates true homogeneity, whereas the value 25, 50, and 75% represented low, moderate and high heterogeneity, respectively. Subgroup analysis was done by the study country, study design, and year of publication. Sensitivity analysis was employed to examine the effect of a single study on the overall estimation. Publication bias was checked by the funnel plot and more objectively through Egger’s regression test.

## Results

**Fig. 1:**
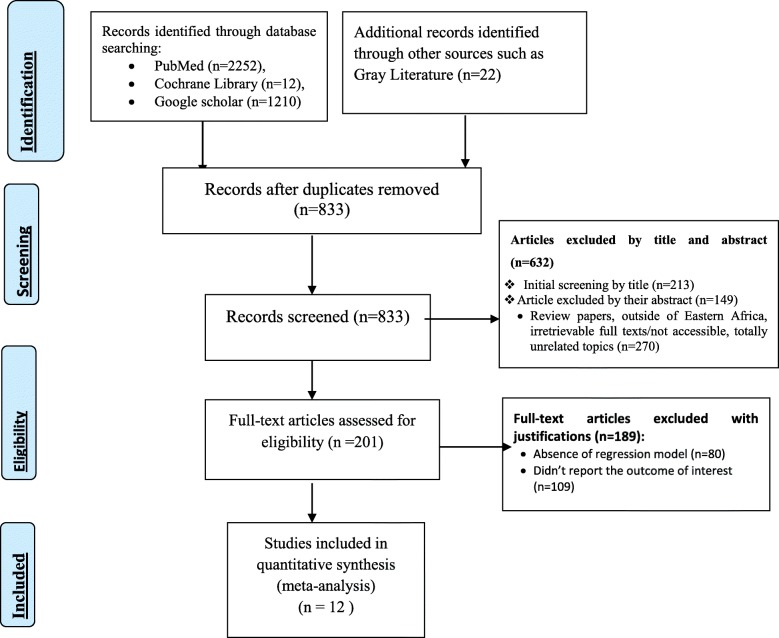
PRISMA –adapted flow diagram showed the results of the search and reasons for exclusion [20, 21]

A total of 3496 studies were identified; 2252 from PubMed, 12 from Cochrane Library, 1210 from Google Scholar and 22 from other sources. After duplication removed, a total of 833 articles remained (2663 removed by duplication). Finally, 201 studies were screened for full-text review, and 12 articles with (*n* = 20,911 patients) were selected for the prevalence and/ or associated factors analysis (Fig. 1, Table S2, and Table S3).

### Characteristics of included studies

Table 1 summarizes the characteristics of the 12 included studies in this systematic review [10, 14, 18, 22–30] Eight studies were found in Ethiopia [10, 18, 23–28], 2 in Kenya [29, 30], while 2 were from Uganda [14, 22]. Nine studies were cross-sectional, while the others used either case-control (*n* = 1) or cohort (*n* = 2) study design. Most of the studies, 8/12(66.7%) were published between 2010 and 2017. The total number of participants in the included studies ranging from 136 [30] to 15,191 [29] (Table 1).

**Table 1:** Distribution of included studies on the prevalence and determinants of neonatal hypothermia in East Africa, from January 2000–December 2019

Author	year	Country	Study design	Sample size	Prevalence (%)	Type of study	Definition of hypothermia	Study outcome
Byaruhanga R et al [14]	2005	Uganda	cross-sectional	300	79	Hospital-based	Axillary temperatures < 36.5 °C	Prevalence at admission (postnatal ward)
Bergstrom A et al [22]	2005	Uganda	case-control	249	46	Hospital-based	Axillary temperatures < 36.5 °C	Prevalence at admission (postnatal ward)
Hayelom G et al [23]	2017	Ethiopia	cross-sectional	1152	53	Hospital-based	Axillary temperatures < 36.5 °C	Prevalence at admission (postnatal ward)
Abayneh G et al [24]	2017	Ethiopia	cross-sectional	769	71	Hospital-based	Axillary temperatures < 36.5 °C	Prevalence at admission (postnatal ward)
Birhanu W et al. [10]	2018	Ethiopia	cross-sectional	356	64	Hospital-based	Axillary temperatures < 36.5 °C	Prevalence at admission (postnatal ward)
Gebresilasea G et al. [25]	2019	Ethiopia	cross-sectional	354	50.3	Hospital-based	Axillary temperatures < 36.5 °C	Prevalence at admission (postnatal ward)
Tewodros S et al [26]	2015	Ethiopia	cohort	421	69.8	Hospital-based	Axillary temperatures < 36.5 °C	Prevalence at admission (postnatal ward)
Hagos T et al [27]	2017	Ethiopia	cross-sectional	264	???	Hospital-based	Axillary temperatures < 36.5 °C	Prevalence at admission (postnatal ward)
Wubet A et al [28]	2019	Ethiopia	cross-sectional	403	66.3	Hospital-based	Axillary temperatures < 36.5 °C	Prevalence at admission (postnatal ward)
Mekonnen T et al [18]	2018	Ethiopia	cross-sectional	1316	13	Hospital-based	Axillary temperatures < 36.5 °C	Prevalence at admission (postnatal ward)
Talbert A et al [29]	2009	Kenya	cohort	15,191	–	Hospital-based	Axillary temperatures < 36.5 °C	Prevalence at admission (postnatal ward)
Switchenko N et al [30]	2017	Kenya	cross-sectional	136	60	Hospital-based	Axillary temperatures < 36.5 °C	Prevalence at admission (postnatal ward)

### Meta-analysis

#### Prevalence of neonatal hypothermia

**Fig. 2:**
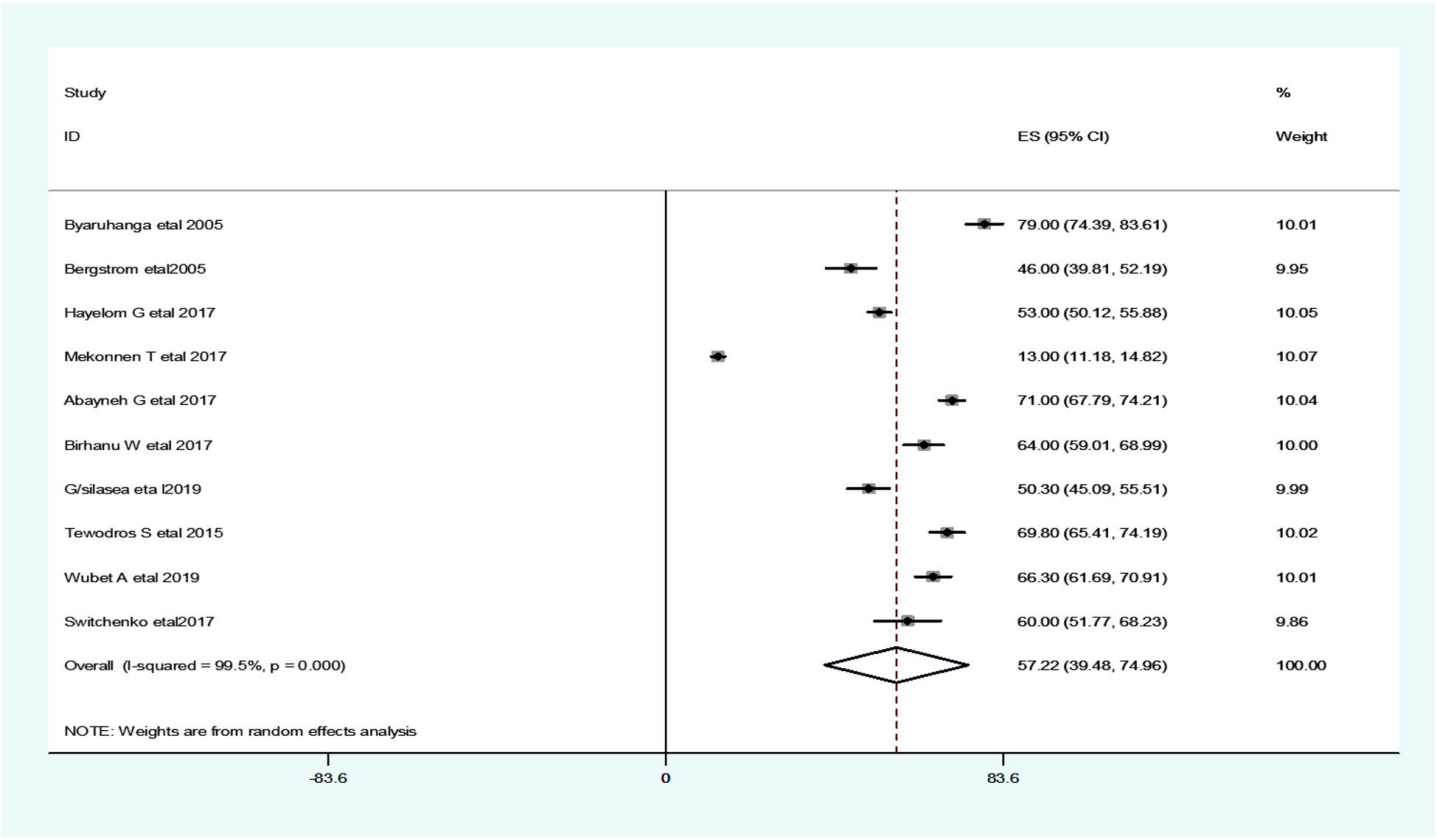
Forest plot showing the prevalence of neonatal hypothermia in East Africa

Most of the studies (*n* = 10) have reported the prevalence of neonatal hypothermia [10, 14, 18, 22–26, 28, 30]. The prevalence of hypothermia was ranged from 13% [18] to 79% [14]. The random-effects model analysis from those studies revealed that, the pooled prevalence of neonatal hypothermia in East Africa was found to be 57.2% (95% CI; 39.48–74.95; I^2^ = 99.5%; *p* < 0.001) (Fig. 2).

#### Subgroup analysis of the prevalence of neonatal hypothermia in eastern Africa

**Fig. 3:**
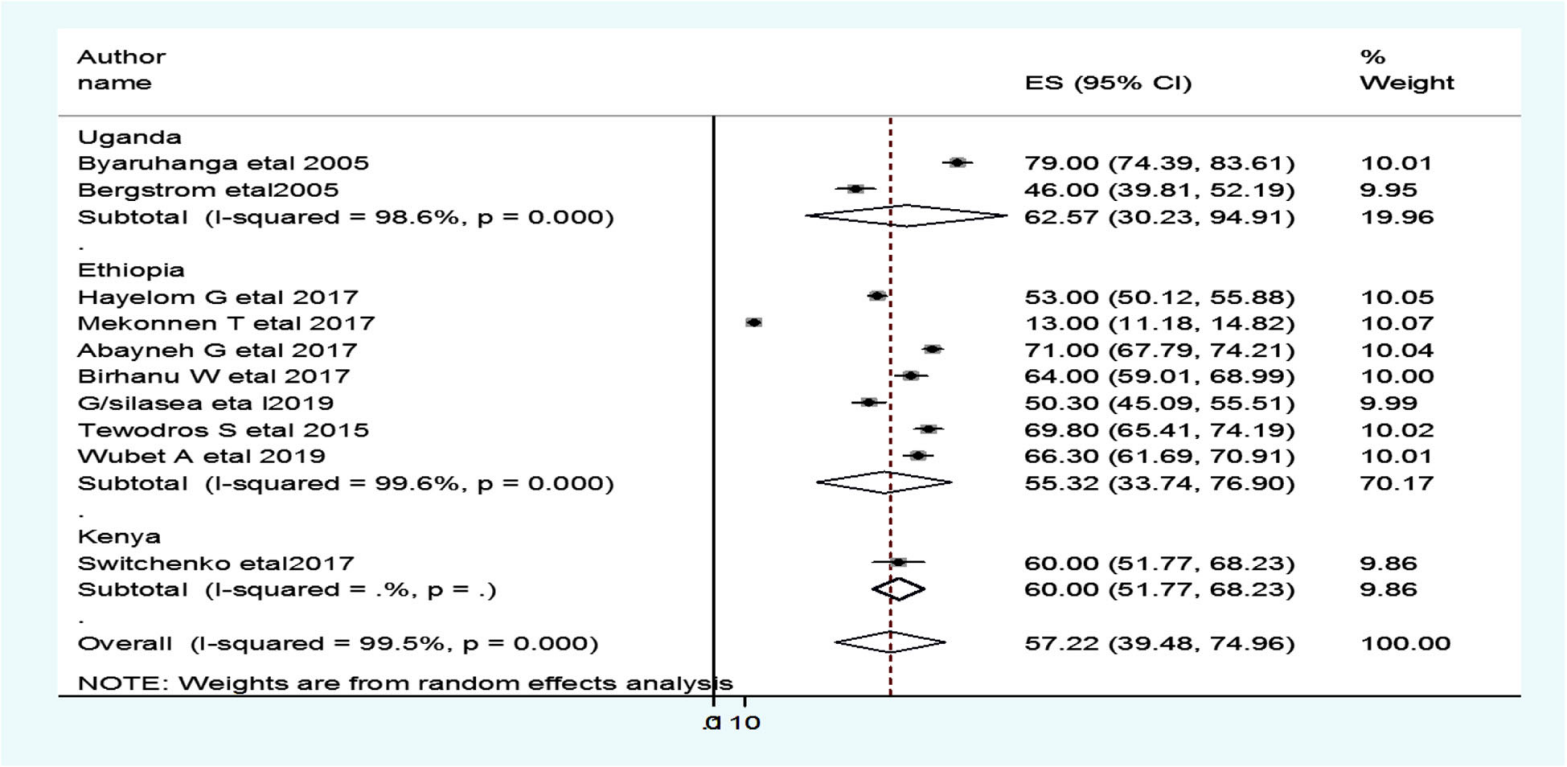
Forest plot showing the subgroup analysis of the prevalence of neonatal hypothermia by country

**Fig. 4:**
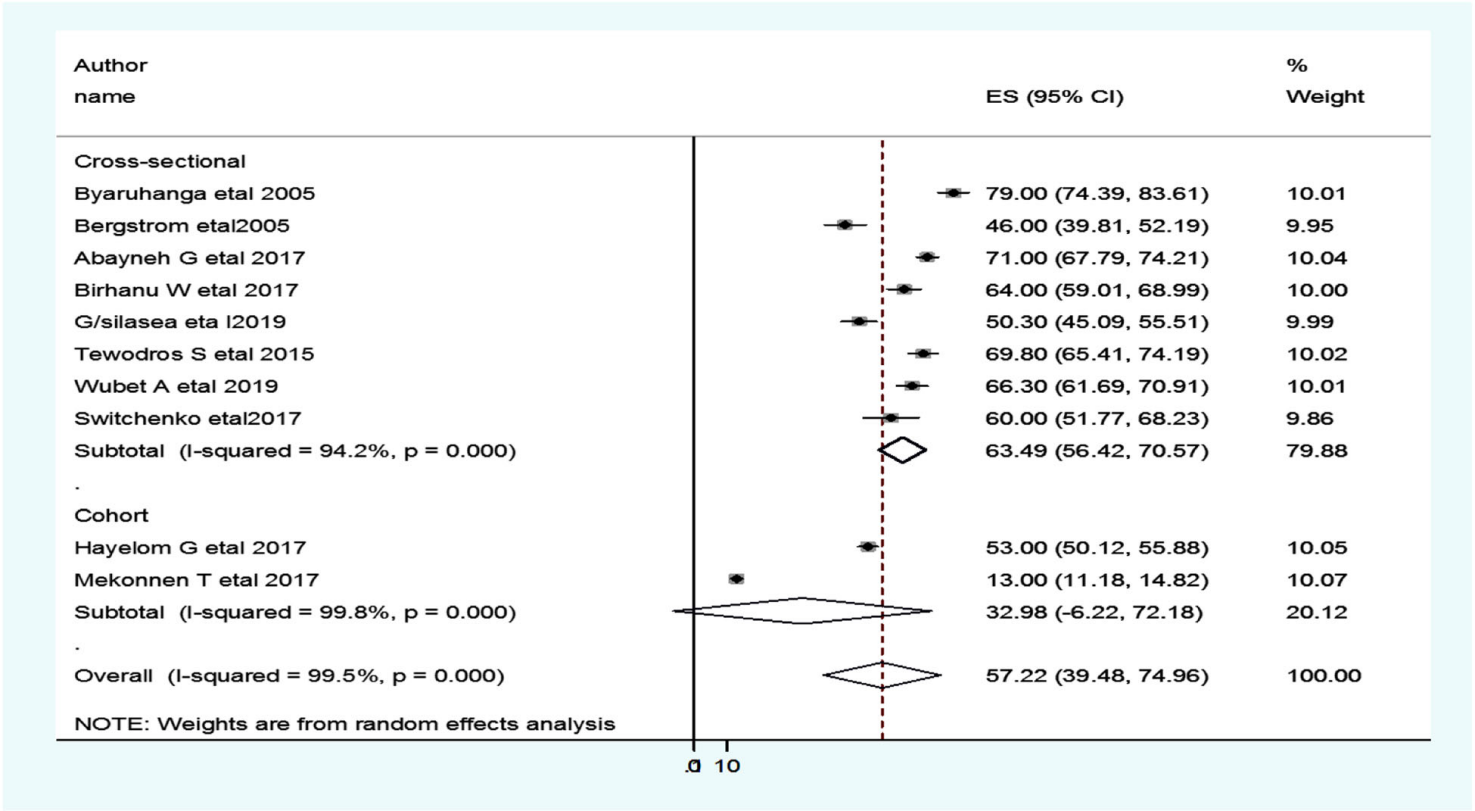
Forest plot showing the subgroup analysis of the prevalence of neonatal hypothermia by study design

**Fig. 5:**
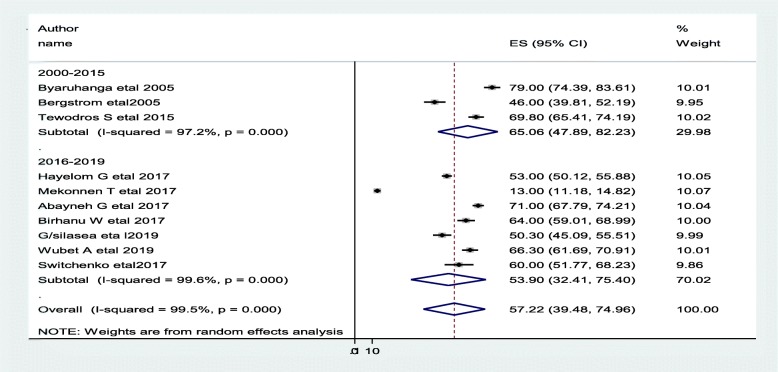
Forest plot showing the subgroup analysis of the prevalence of neonatal hypothermia by year of publication

The subgroup analysis was done through stratified by country, study design, and year of publication. Based on this, the prevalence of neonatal hypothermia was found to be 55.3% in Ethiopia, 62.6% in Uganda, and 60.0% in Kenya (Fig. 3 and Table 2). Based on the study design, the prevalence of neonatal hypothermia was found to be 63.5% in cross-sectional studies and 32.98% in cohort studies (Fig. 4 and Table 2). Based on the year of publication, the prevalence of neonatal hypothermia was found to be 65.1% from studies conducted from January 2000–December 2015, while it was 57.9% from studies conducted from 2016 to 2019(Fig. 5 and Table 2).

**Table 2:** Summary of subgroup analysis of the prevalence of neonatal hypothermia in Eastern Africa by country, design and year of publication, from January 2000–December 2019

Variables	Characteristics	Pooled prevalence, %(95% CI)	I^**2**^, (***P-value***)
By country	Ethiopia	55.3 (33.7–76.9)	99.6%(< 0.001)
	Uganda	62.6 (30.2–94.9)	98.6%(< 0.001)
	Kenya	60.0 (51.8–68.2)	99.5%(< 0.001)
By study design	Cross-sectional	63.5 (56.4–70.6)	94.2% (< 0.001)
	Cohort	33.0 (6.2–72.2)	99.8%(< 0.001)
By year of publication	2000–2015	65.1 (47.9–82.2)	97.2% (< 0.001)
	2016–2019	57.9 (32.4–75.4)	99.6%(< 0.001)

### Publication bias

A funnel plot showed asymmetrical distribution. The Egger’s regression test*-value* was 0.019, which indicated that, the presence of publication bias. Due to the presence of publication bias, we employed a leave-one-out sensitivity analysis to identify the potential source of heterogeneity in the analysis of the prevalence of neonatal hypothermia in Eastern Africa. The results of this sensitivity analysis showed that the findings were not dependent on a single study. Our pooled estimated prevalence of neonatal hypothermia varied from 54.8% (36.5–73.1) to 62.3% (55.2–69.3) after the deletion of a single study. Two studies, Byaruhanga R, 2005 [14] Mekonnen T, 2018 [18] had shown an impact on the overall estimation.

### Factors associated with neonatal hypothermia in eastern Africa

#### Delayed initiation of breastfeeding

Timely initiation of breastfeeding is considered as initiating breastfeeding within 1 hour after birth. Five studies found a significant association between delayed initiation of breastfeeding and neonatal hypothermia [10, 25–28]. The odd of neonatal hypothermia among newborns with delayed initiation of breastfeeding range from 1.63 [28] to 4.39 [10] (Table 3).

**Table 3:** Identified associated factors for neonatal hypothermia from studies in East Africa, January 2000–2019

Determinants	Odds ratio (aOR, 95% CI)	Author	Year of publication	Reference
Delay in the initiation of breastfeeding	4.39 (2.38, 8.11)	Birhanu W et al	2018	[10]
	2.42 (1.45, 4.02)	Gebresilasea et al	2019	[25]
	7.58 (3.61,15.91)	Tewodros et al	2015	[26]
	7.23 (2.75,18.99)	Hagos et al	2018	[27]
	1.63 (0.88,2.99)	Wubet et al	2019	[28]
Neonatal health problem	3.65 (1.83,8.44)	Birhanu W et al	2018	[10]
	2.46 (1.64,8.18)	Gebresilasea et al	2019	[25]
	3.10 (1.06, 9.46)	Tewodros et al	2015	[26]
	2.28 (0.64,8.18)	Hagos et al	2018	[27]
	4.24 (1.92,9.34)	Wubet et al	2019	[28]
Low birth weight	1.33 (0.75,2.36)	Birhanu et al	2018	[10]
	3.61 (2.1,6.18)	Gebresilasea et al	2019	[25]
	3.75 (1.29,10.88)	Tewodros et al	2015	[26]
	8.51 (2.71,26.73)	Hagos et al	2018	[27]
	1.20 (0.51,2.82)	Wubet et al	2019	[28]
Preterm	4.81 (2.67,8.64)	Birhanu et al	2018	[10]
	4.61 (2.83,8.39)	Gebresilasea et al	2019	[25]
	1.50 (0.84,0.26)	Tewodros et al	2015	[26]
	3.69 (1.36,10.01)	Hagos et al	2018	[27]
	3.37 (1.53,7.44)	Wubet et al	2019	[28]
Nighttime delivery	1.32 (0.73,2.37)	Birhanu et al	2018	[10]
	1.68 (1.01,2.83)	Gebresilasea et al	2019	[25]
	6.61 (3.75,11.66)	Tewodros et al	2015	[26]
	6.25 (2.58,15.12)	Hagos et al	2018	[27]
	3.18 (1.28,4.57)	Wubet et al	2019	[28]

**Fig. 6:**
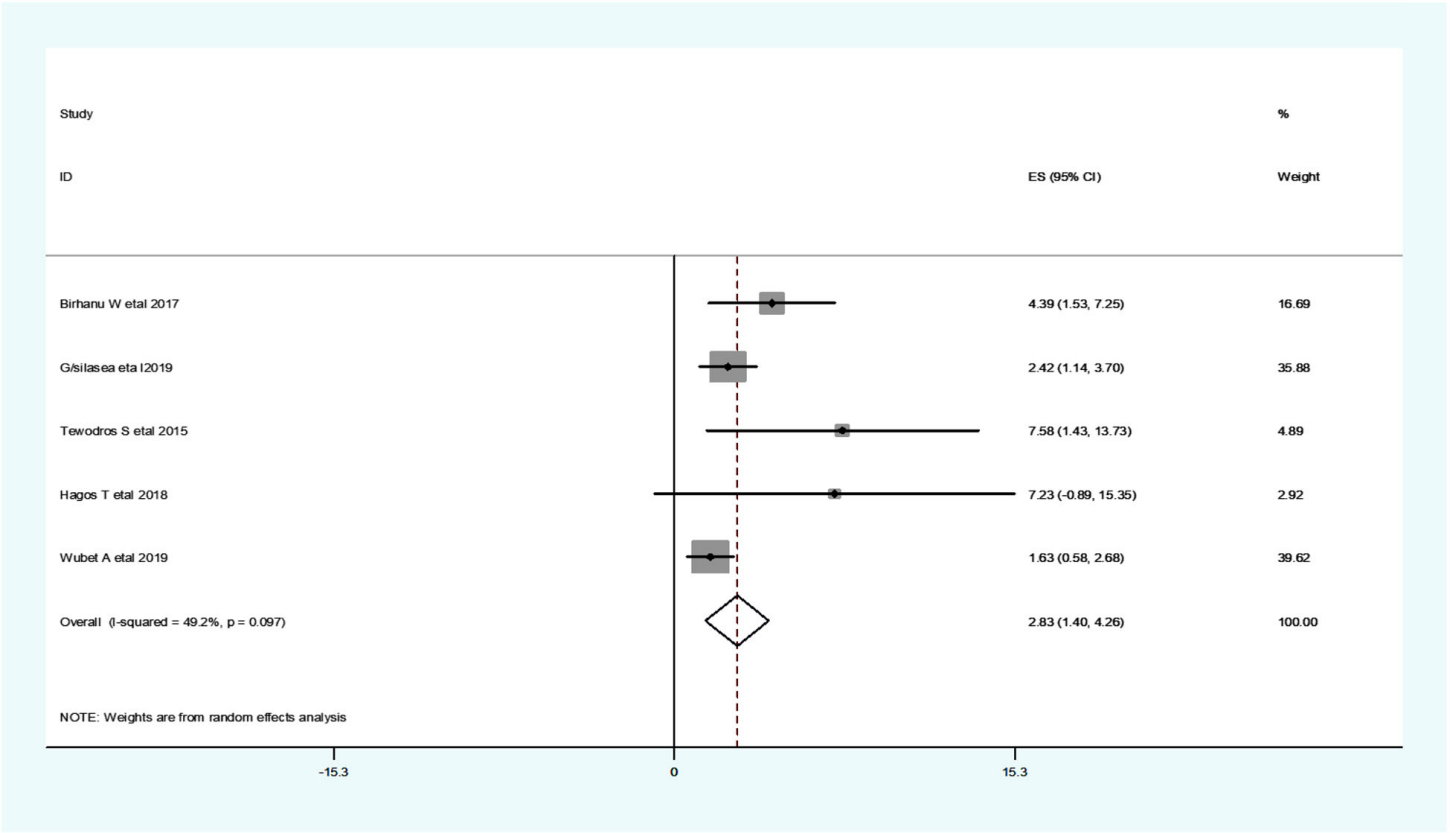
Forest plot showing a pooled estimate of delayed initiation of breastfeeding

***Regarding heterogeneity test***, the Galbraith plot showed homogeneity and combining the result of five studies, the forest plot showed the overall estimate of delayed initiation of breastfeeding was, **aOR** = 2.83(95% CI: 1.398–4.26;I^2^ = 49.2%;*P* = 0.097).I-Squared (I^2^) and *P-value* also showed homogeneity (Fig. 6).

***Regarding publication bias***, a funnel plot showed an asymmetrical distribution. During the Egger’s regression test, the *p-value* was 0.016, which indicated the presence of publication bias. Hence, trim and fill analysis was done, and 2 studies were added, and the total number of studies becomes seven. The pooled estimate of **aOR** of delayed initiation of breastfeeding was found to be 2.463.

#### Neonatal health problems

Neonatal health problems refer to a presentation of the neonate with any problem that can trouble its health (congenital malformation, asphyxia, jaundice, respiratory distress, bleeding disorder, meconium aspiration syndrome) [28].

In our analysis, five studies found a significant association between neonatal health problems and neonatal hypothermia [10, 25–28]. The odd of neonatal hypothermia among newborns with neonatal health problems range from 2.28 [27] to 4.24 [28] (Table 3).

**Fig. 7:**
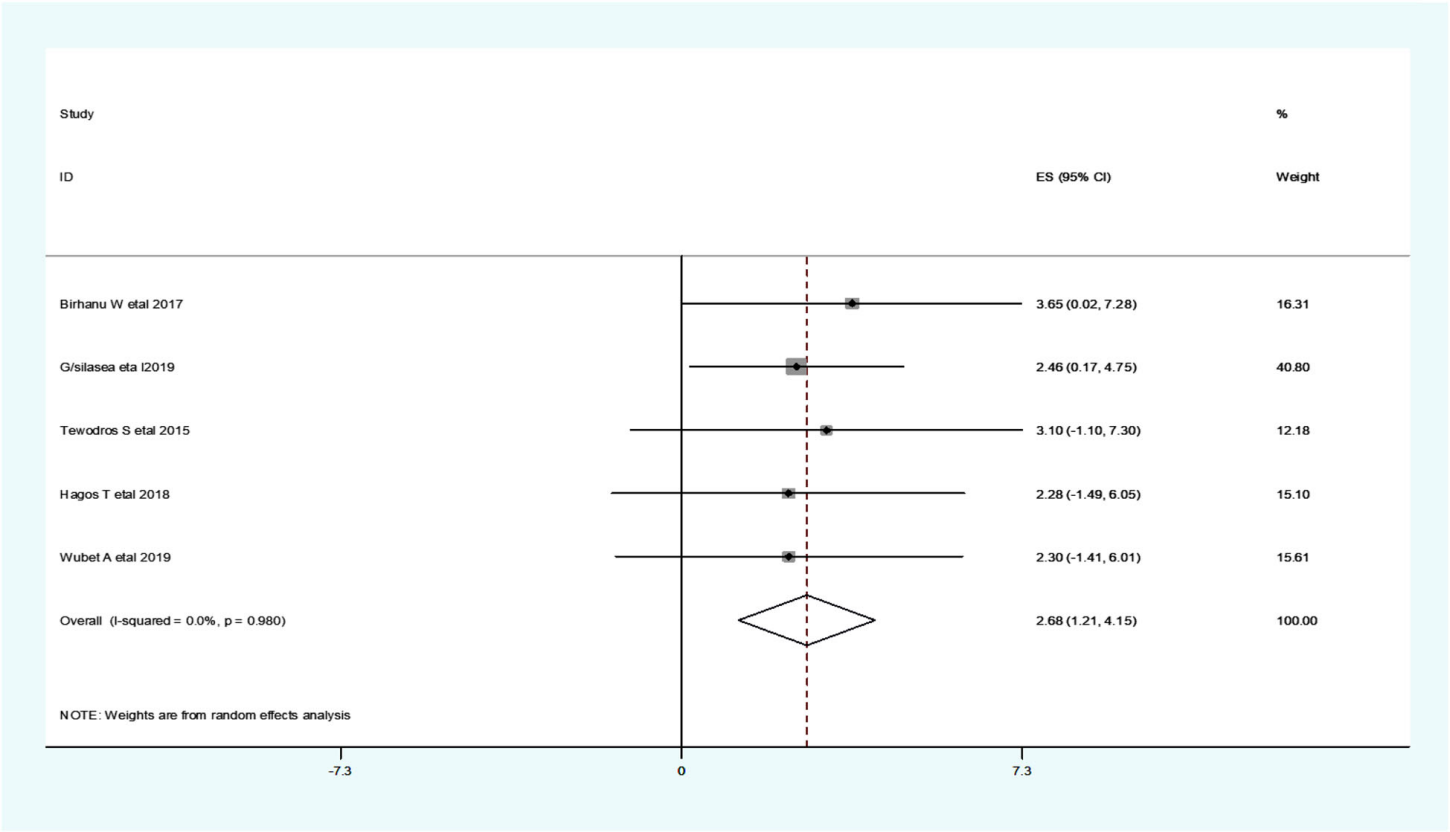
Forest plot showing a pooled estimate of neonatal health problems in East Africa

Regarding the heterogeneity test for neonatal health problems, the Galbraith plot showed homogeneity and combining the result of five studies, the forest plot showed the overall estimate of neonatal health problems was, **aOR** = 2.68(95% CI: 1.21–4.15;I^2^ = 0.0%;*P* = 0.98).I-Squared (I^2^) and *P*-value also showed homogeneity (Fig. 7).

Regarding the publication of bias for neonatal health problems analysis, the funnel plot analysis showed asymmetrical distribution. During the Egger’s regression test, the *p*-value was 0.068, which indicated the absence of publication bias. Hence, trim and fill analysis was done, and 1 study was added, and the total number of studies become six. The pooled estimate of **aOR** of neonatal preterm becomes 2.49.

We employed a leave-one-out sensitivity analysis to identify the potential source of heterogeneity in the analysis of the prevalence of neonatal hypothermia in Eastern Africa. The results of this sensitivity analysis showed that the findings were not dependent on a single study. Our pooled estimate of neonatal health problems varied from 2.49(95%CI, 0.88–4.09) to 2.75(95% CI, 1.15–4.34) after the deletion of a single study.

#### Low birth weight

Low birth weight was considered when the neonate’s birth weight is less than 2.5 kg. Five studies found a significant association between neonate’s low birth weight and hypothermia [10, 25–28]. The odd of neonatal hypothermia among low birth weight neonates range from 1.33 [10] to 8.51 [27] (Table 3).

**Fig. 8:**
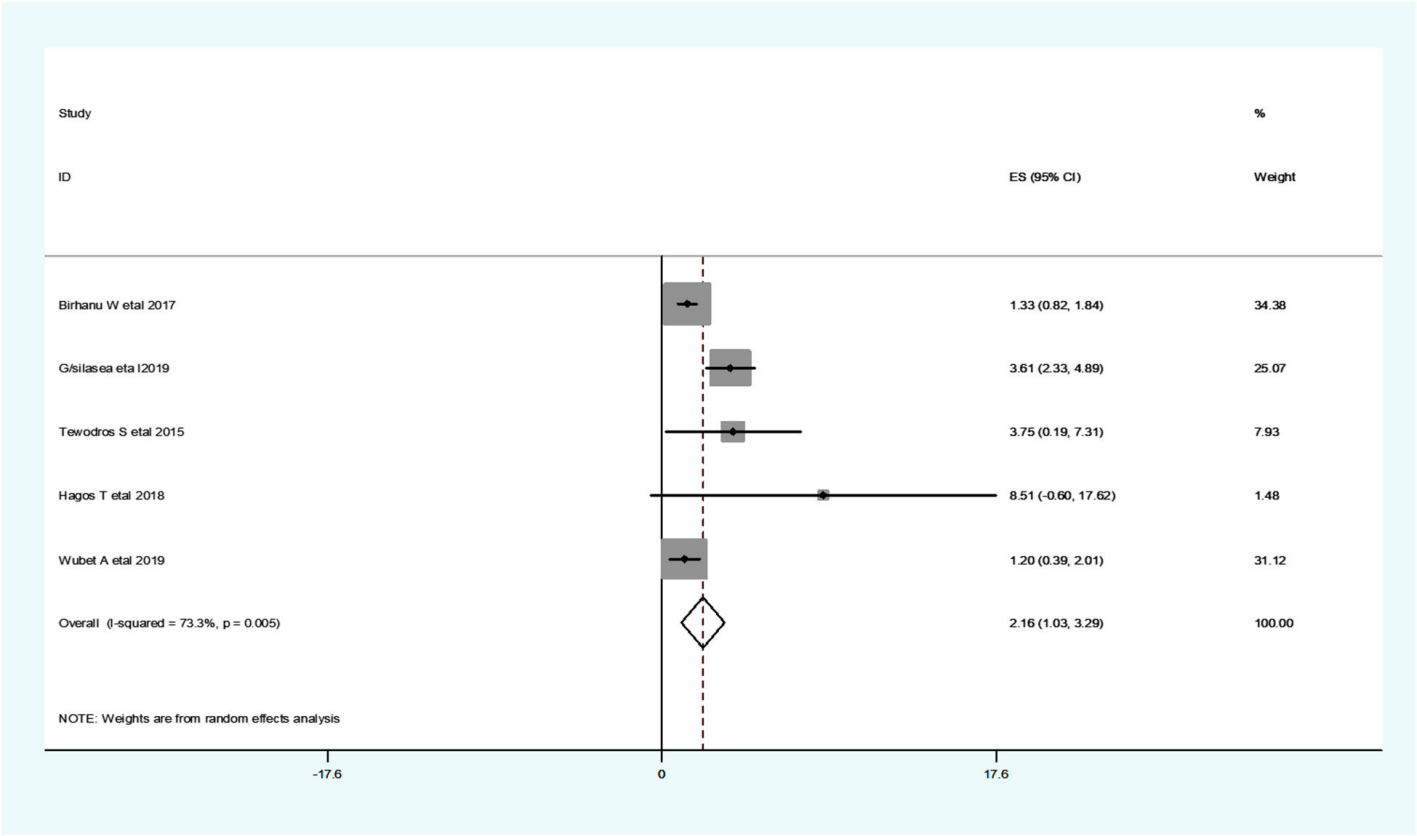
Forest plot showing the pooled estimate of low birth weight

Regarding heterogeneity test, the Galbraith plot showed heterogeneity and combining the result of five studies, the forest plot showed the overall estimated **aOR** of low birth weight was 2.16(95%CI: 1.027–3.293;I^2^ = 3.3%;*P* = 0.005).I-Squared (I^2^) and *P*-value also showed heterogeneity (Fig. 8).

*Regarding publication bias*, a funnel plot showed a symmetrical distribution. During the Egger’s regression test, the *p*-value was 1.98, which indicated the absence of publication bias. Trim and fill analysis was done, and 2 studies were added, and the total number of studies become seven. The pooled estimated OR of neonate’s low birth weight becomes 1.85.

#### Preterm

Preterm was considered when the delivery is less than 37 weeks of gestational age. Five studies found a significant association between preterm and neonatal hypothermia [10, 25–28]. The odd of neonatal hypothermia among preterm neonates range from 1.5 [26] to 4.81 [10] (Table 3).

**Fig. 9:**
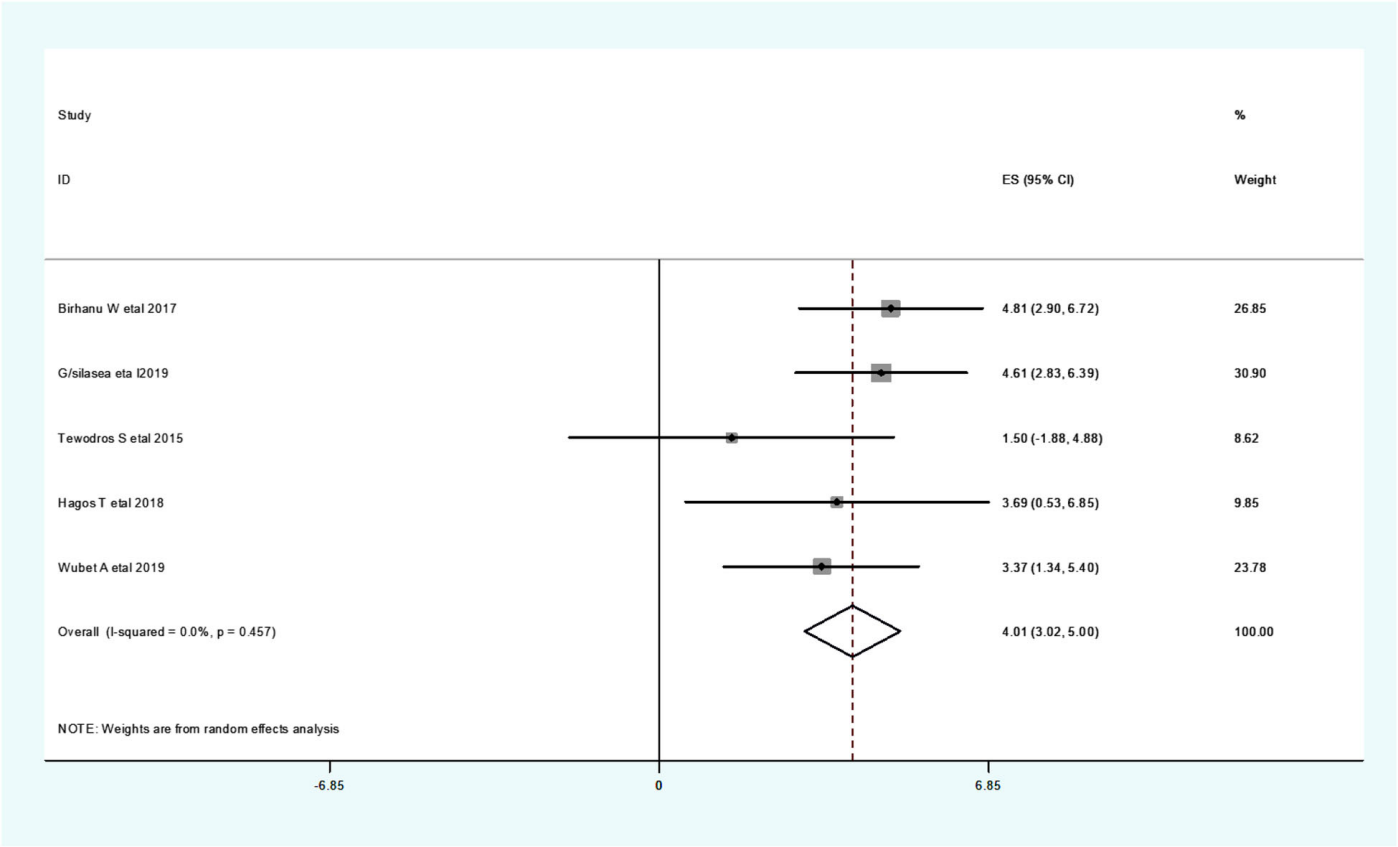
Forest plot showing the pooled estimate of preterm

Regarding heterogeneity test, the Galbraith plot analysis showed homogeneity and combining the result of five studies, the forest plot showed the overall estimate of **aOR** of preterm was 4.01(95%C I: 3.02,5.00;I^2^ = 0.0%;*P* = 0.457).I-Squared (I^2^) and *P*-value also showed homogeneity (Fig. 9).

Regarding publication bias, a funnel plot showed a symmetrical distribution. During Egger’s regression test, the *p*-value was 0.131, which indicated the presence of publication bias.

#### Nighttime delivery

Five studies found a significant association between nighttime delivery and neonatal hypothermia [10, 25–28]. The odd of neonatal hypothermia among neonates who delivered at night range from 1.32 [10] to 6.25 [27] (Table 3).

**Fig. 10:**
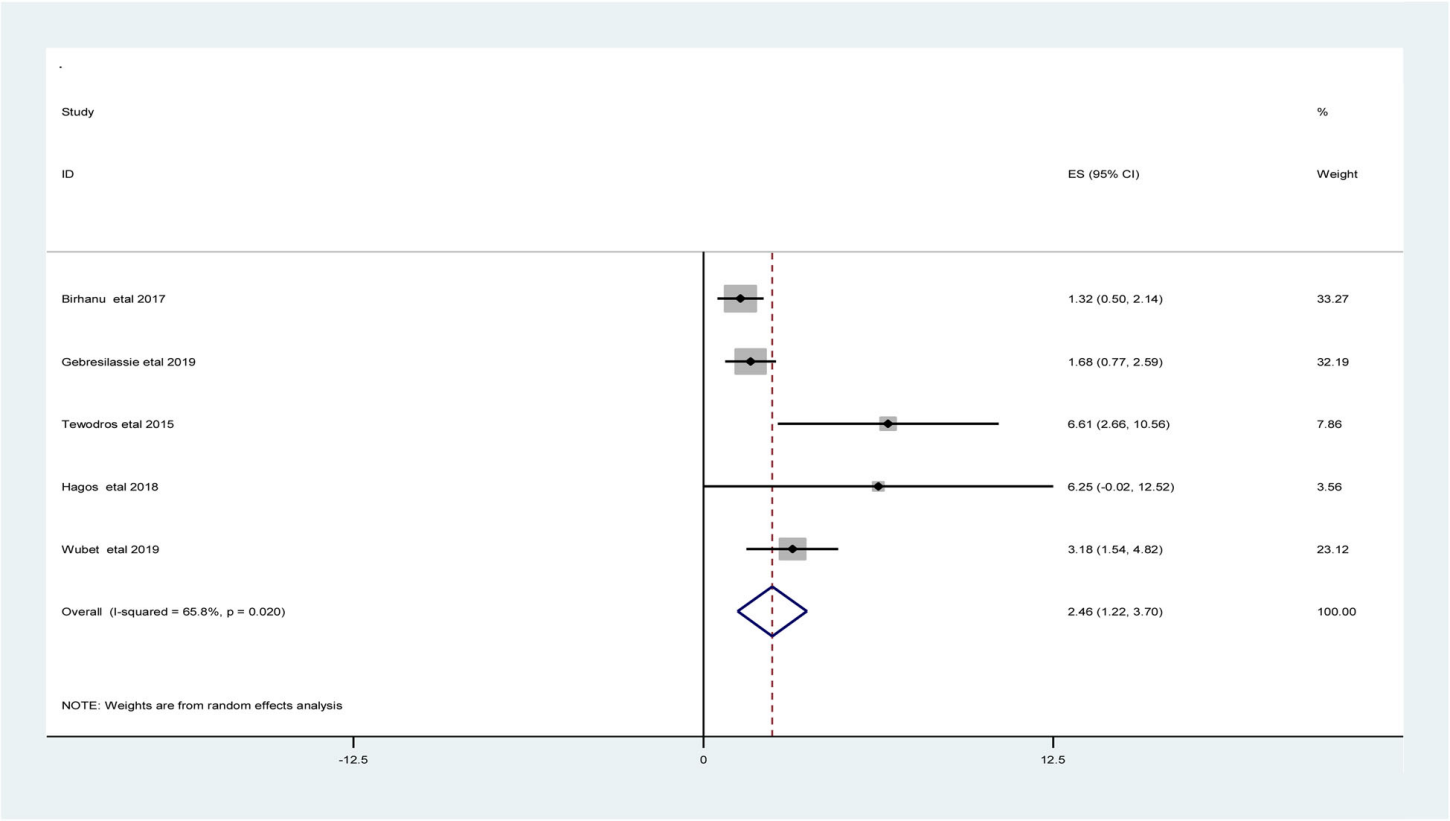
Forest plot showing the pooled estimate of nighttime delivery of neonates in East Africa, 2000–2019

Regarding heterogeneity test, the Galbraith plot showed homogeneity and combining the result of five studies, the forest plot showed the overall estimate **aOR** of nighttime delivery was 2.46 (95% CI: 1.22–3.70;I^2^ = 65.8%;*P* = 0.020).I-Squared (I^2^) and P-value also showed heterogeneity (Fig. 10).

Regarding publication bias, the funnel plot analysis showed a symmetrical distribution. During the Egger’s regression test, the *p*-value was 0.131, which indicated the absence of publication bias.

## Discussion

In this systematic review and meta-analysis, we explored the prevalence and determinants of neonatal hypothermia in Eastern Africa. In total, 12 studies judged to be of low risk of bias were included in the final analysis. Based on the analysis a significant proportion (more than 1 in 2) of neonates had neonatal hypothermia in Eastern Africa. This shows that neonatal hypothermia is a significant public health problem in Eastern Africa. We also identified factors that were significantly associated with neonatal hypothermia in Eastern Africa. In this study, the pooled prevalence of neonatal hypothermia in Eastern Africa was 57.22% (95%CI; 39.48–74.95). The results of this meta-analysis were in line with other systematic review findings reported from Nigeria [31], Bahir Dar, Ethiopia [32], Northwest Ethiopia [33], a review conducted in sub Saharan Africa [3]. According to another meta-analysis conducted on the global burden of neonatal hypothermia the prevalence of hypothermia was ranged from 32 to 85% [4].

However, the results of this meta-analysis were higher than the review conducted in Iran which was ranged from 7.48 to 53.3% [34]*,* a study conducted in Bangladesh [35], Pakistan [36], and South Africa [37]. In contrast, the magnitude was lower than a study conducted in Zimbabwe [4], Nepal [38], and Uganda [14]. This discrepancy might be due to the socioeconomic and cultural variation between the countries, and study settings. Moreover, the other obvious reason for the difference might be due to the sample size, a collection of data from different settings (community and health institutions) as well as different study periods. Furthermore, this variation might be due to the difference in temperature measurement site, ecological, economic and cultural difference between the study areas.

Several physiological, behavioral, environmental and socioeconomic factors can increase the odds of neonatal hypothermia. This study showed that delay in initiation of breastfeeding, having neonatal health problems, neonate’s low birth weight, being preterm, and nighttime delivery was identified factors that significantly raise the risk of neonatal hypothermia. Similar findings were also reported from previous meta-analysis studies [39–41].

### Physiological factors

The odds of hypothermia were higher among preterm neonates when compared to term neonates. The possible reason might be preterm neonates skin is immature and thin that increases loss of heat through radiation. Inadition they have immature hypothalamic thermal control, and they have insufficent neural mechanisms for temperature control by shivering, have low glycogen stores and adipose tissue. Hence, they have decreased ability to regulate their body temperature, by producing heat through non-shivering thermogenesis [3].

The odds of hypothermia were higher among neonates with low birth weight when compared to those who had normal birth weight. This is consistent with a study done in Northwest Ethiopia, Nigeria, and Pakistan [13, 33, 36]. This is also supported by the findings from studies conducted in Iran where low birth weight had increased risk of neonatal hypothermia by more than four fold, and another Iranian study revealed that LBW increases the risk of hypothermia by 2.5-fold [36, 42]. Other previous studies conducted in Nepal [5], and Nigeria [13] reported the same finding that low birth weight had increased risk of neonatal hypothermia by 1.5-fold. The study conducted in Ethiopia reported that LBW had increased the risk of hypothermia by 3.7-fold [33]. This is because babies with small weight have a large surface area per unit of body weight which makes them prone to develop hypothermia [33]. The other reason is, low birth weight babies have decreased thermal insulation due to less subcutaneous fat and the reduced amount of brown fat [43].

### Behavioral factors

The odds of hypothermia were higher among neonates with delayed initiation of breastfeeding as compared to those who had started breastfeeding within 1 hour after birth. This might be due to the reason that breast milk is the source of energy or calories to produce heat for thermoregulation and they have no adequate adipose tissue for glucose breakdown which results in hypothermia [44]. Besides skin-to-skin contact is an external heat source for the baby during breastfeeding. This finding is lower than the finding reported in Ethiopia [13, 33]. This difference in magnitude might be due to the difference in study setup, knowledge of mothers on good positioning and attachment of breastfeeding and difference in place of delivery. Besides, early bathing contributes significantly to heat loss and increases the incidence of hypothermia in cold climates [25], and should be postponed until at least after the first 6 h of life, and possibly longer.

### Environmental factors

Babies who born during the night were more likely to develop hypothermia as compared to neonates who born during day time. A similar finding has been reported from other study in Ethiopia [26]. This might be because room temperature is low during the night as compared to day time. It also is, due to the work overload during night time as the number of healthcare workers working in the labour room during the night is not equal to day time staff.

### Socioeconomic factors

An infant’s low body temperature is also associated with having a young and inexperienced mother, coming from a family with low socioeconomic status, or being born to a mother who already had multiple births. While some of these physiologic risk factors have been documented decades ago, awareness of the risks associated with hypothermia, as indicated in a multinational survey and another one from India, indicating that health care professionals have limited knowledge of the diagnosis and management of newborn hypothermia [13, 14]**.** The following strategies should be implemented to reduce the prevalence of neonatal hypothermia: early initiation of breastfeeding, education of staff and mothers, warm chain, drying, wrapping, and quality improvement [45–47]. This study has several strengths: First, all included studies are of low risk of bias after we conducted quality assessment using a standardized JBI checklist. Second, we used a pre-specified protocol for search strategy, and data abstraction; we also employed subgroup and sensitivity analysis based on study country, study design, and publication year to identify the small study effect and the risk of heterogeneity. Nevertheless, this review had some limitations: we found studies that fulfill the inclusion criteria and have a low risk of bias in only 3 of 7 East African countries and are represented with 8 of 12 included studies conducted in Ethiopia. Besides, the result in this meta-analysis is derived from studies conducted in hospital settings, and this limits the generalizability of the review findings; since, it had not included community-based studies. In addition, there may be publication bias because not all grey literature are included and language bias; since all included studies are published in English.

## Conclusions

The prevalence of neonatal hypothermia in Eastern Africa remains high. Delay in initiation of breastfeeding, having a neonatal health problem, being low birth weight, preterm, and nighttime delivery were identified factors that significantly increase the risk of neonatal hypothermia. It is recommended that early initiation of breastfeeding should be promoted, and emphasis should be given towards low birth weight, preterm and neonates with neonatal problems to prevent burdens of hypothermia in East Africa. This review may help policymakers and program officers to design neonatal hypothermia preventive interventions. The identified gaps in these studies are: To the best of our knowledge, there is limited information on neonatal hypothermia from some of Eastern Africa countries. Since, in most parts of the Eastern African countries, the temperature is not measured and recorded in most newborns immediately after birth, the epidemiological picture of hypothermia and its clinical consequences is yet incomplete. This implies additional research should be done in most of Eastern Africa countries with standard measurements of body temperature using a better design like Randomized Control Trial. In addition methodologically sound hospital-based and community-based studies are required to understand the problem in Eastern Africa settings. Attention is needed for the thermal care of newborns and the use of low-cost thermal protection principles especially for those preterm, low birth weight and newborns with health problems during early initiation of breastfeeding immediately after delivery. It is also important to give attention to babies delivered during nighttime. Moreover, increase awareness/ education of health professionals and mothers of risks of hypothermia and thermal care measures such as the warm chain including skin-to-skin/ kangaroo care.

## Supplementary information

**Additional file 1: Table S1.** Search strategy used for one of the databases.

**Additional file 2: Table S2.** Quality appraisal result of included studies in East Africa, from January 2000–December 2019.; Using Joanna Briggs Institute (JBI) quality appraisal checklist [16].

**Additional file 3: Table S3.** Adjusted confounders and main findings extracted from included studies in East Africa, from January 2000–December 2019.

## Data Availability

Data is available and it can be accessed from the corresponding author with a reasonable inquiry.

## Abbreviations

WHO: World Health Organization
CI: Confidence interval
aOR: adjusted Odds Ratio
ENBC: Essential newborn care
LBW: low birth weight
NMRs: Neonatal mortality rates
SDG: Sustainable development goal
PRISMA: Preferred Reporting Items for Systematic Reviews and Meta-Analyses
