# Supplementary material for: Prevalence of neonatal hypothermia and its associated factors in East Africa: a systematic review and meta-analysis
Source: BMC Pediatr. 2020 Apr 3;20:148. doi: 10.1186/s12887-020-02024-w (PMC7118870; doi:10.1186/s12887-020-02024-w)
Supplement: Supplementary file 1 — Additional file 1: Table S1. Search strategy used for one of the databases. [file 12887_2020_2024_MOESM1_ESM.docx]

# Table S1. Search strategy used for one of the databases

| Medline/PubMed | | |  |
| --- | --- | --- | --- |
|  | **Search terms** | |  |
| Group | **Non-MeSH terms** | **MeSH (sub-terms in MeSH)** | **Citations** |
| #1 | Prevalence  Magnitude |  |  |
| #2 | Causes  Determinants  Associated factors  Predictors |  |  |
| #3 | Neonate  Infant  Child  Children | Newborn |  |
| #4 | Low body temperature  Thermoregulation  Body temperature regulation | Hypothermia |  |
| #5 | Eastern Africa  Developing country |  |  |
| #1 AND #2 AND #3 AND #4 AND #5 |  |  | **2252** |

(Prevalence OR magnitude) AND (causes OR determinants OR associated factors OR predictors) AND (newborn [MeSH Terms] OR neonate OR infant OR child OR children) AND (hypothermia [MeSH Terms] OR low body temperature OR thermoregulation OR body temperature regulation)AND (Eastern Africa) OR developing country.
