# Supplementary material for: Prevalence of neonatal hypothermia and its associated factors in East Africa: a systematic review and meta-analysis
Source: BMC Pediatr. 2020 Apr 3;20:148. doi: 10.1186/s12887-020-02024-w (PMC7118870; doi:10.1186/s12887-020-02024-w)
Supplement: Supplementary file 2 — Additional file 2: Table S2. Quality appraisal result of included studies in East Africa, from January 2000–December 2019.; Using Joanna Briggs Institute (JBI) quality appraisal checklist [16]. [file 12887_2020_2024_MOESM2_ESM.docx]

Table S2: Quality appraisal result of included studies in East Africa, from January 2000-December 2019.; Using Joanna Briggs Institute (JBI) quality appraisal checklist [16],

| **Author** | **Quality assessment questions** | | | | | | | | | | |  |  |  |
| --- | --- | --- | --- | --- | --- | --- | --- | --- | --- | --- | --- | --- | --- | --- |
|  | Q1 | Q2 | Q3 | Q4 | Q5 | Q6 | Q7 | Q8 | Q9 | Q10 | Q11 | Yes Total | Quality status | Overall appraisal |
| Cross-sectional studies | | | | | | | | | | | | | | |
| Byaruhanga R(1) | N | Y | N | Y | Y | N | Y | Y |  |  |  | 5/8 | Low risk | Included |
| Hayelom G /(2) | Y | Y | Y | Y | Y | Y | Y | Y |  |  |  | 8/8 | Low risk | Included |
| Abayneh G /(3) | Y | Y | UC | Y | Y | N | Y | Y |  |  |  | 6/8 | Low risk | Included |
| Birhanu W/(4) | Y | Y | Y | Y | Y | UC | Y | Y |  |  |  | 7/8 | Low risk | Included |
| Gebresilasea G/(5) | Y | UC | Y | Y | Y | N | Y | Y |  |  |  | 6/8 | Low risk | Included |
| Hagos T/(6) | Y | N | Y | Y | Y | UC | Y | Y |  |  |  | 6/8 | Low risk | Included |
| Wubet A /(7) | Y | UC | Y | Y | Y | N | Y | Y |  |  |  | 6/8 | Low risk | Included |
| Mekonnen T/(8) | Y | UC | Y | Y | Y | N | Y | Y |  |  |  | 6/8 | Low risk | Included |
| Switchenko N/(9) | UC | Y | Y | Y | Y | N | Y | Y |  |  |  | 5/8 | Low risk | Included |
| Tewodros S/(10) | Y | Y | Y | Y | Y | N | Y | Y |  |  |  | 7/8 | Low risk | Included |
| **Cohort studies** | | | | | | | | | | | | | | |
| Alison Talbert /(11) | Y | Y | Y | UC | UC | Y | Y | Y | Y | Y | N | 9/11 | Low risk | Included |
| **Case control study** | | | | | | | | | | | | | | |
| Bergstrom A/(12) | Y | Y | Y | UC | Y | Y | N | Y | N | Y |  | 7/10 | Low risk | Included |

Key: Y=yes, N=no, UC=unclear, Q=Question

**References**

1. Byaruhanga R, Bergstrom A, Okong P. Neonatal hypothermia in Uganda: prevalence and risk factors. Journal of tropical pediatrics. 2005;51(4):212-5.

2. Mengesha HG, Sahle BW. Cause of neonatal deaths in Northern Ethiopia: a prospective cohort study. BMC public health. 2017;17(1):62.

3. Demisse AG, Alemu F, Gizaw MA, Tigabu Z. Patterns of admission and factors associated with neonatal mortality among neonates admitted to the neonatal intensive care unit of University of Gondar Hospital, Northwest Ethiopia. Pediatric health, medicine and therapeutics. 2017;8:57.

4. Demissie BW, Abera BB, Chichiabellu TY, Astawesegn FH. Neonatal hypothermia and associated factors among neonates admitted to neonatal intensive care unit of public hospitals in Addis Ababa, Ethiopia. BMC pediatrics. 2018;18(1):263.

5. Ukke GG, Diriba K. Prevalence and factors associated with neonatal hypothermia on admission to neonatal intensive care units in Southwest Ethiopia–A cross-sectional study. PloS one. 2019;14(6):e0218020.

6. Tasew H, Gebrekristos K, Kidanu K, Mariye T, Teklay G. Determinants of hypothermia on neonates admitted to the intensive care unit of public hospitals of Central Zone, Tigray, Ethiopia 2017: unmatched case–control study. BMC research notes. 2018;11(1):576.

7. Bayih WA, Assefa N, Dheresa M, Minuye B, Demis S. Neonatal hypothermia and associated factors within six hours of delivery in eastern part of Ethiopia: a cross-sectional study. BMC pediatrics. 2019;19(1):252.

8. Mekonnen T, Tenu T, Aklilu T, Abera T. Assessment of Neonatal Death and Causes among Admitted Neonates in Neonatal Intensive Care Unit of Mizan Tepi University Teaching Hospital, Bench Maji Zone, South-West Ethiopia, 2018. Clinics Mother Child Health. 2018;15(305):2.

9. Switchenko N KE FB. Prevalence of neonatal hypothermia in a referal hospitals newborn unit in Kenya. . 2017.

10. Ebrahim TSaE. Proportion of Neonatal Hypothermia and Associated Factors among New-borns at Gondar University Teaching and Refferal Hospital, Northwest Ethiopia: A Hospital Based Cross Sectional Study.

11. Talbert A, Atkinson S, Karisa J, Ignas J, Chesaro C, Maitland K. Hypothermia in children with severe malnutrition: low prevalence on the tropical coast of Kenya. Journal of tropical pediatrics. 2009;55(6):413-6.

12. Bergström A, Byaruhanga R, Okong P. The impact of newborn bathing on the prevalence of neonatal hypothermia in Uganda: a randomized, controlled trial. Acta Paediatrica. 2005;94(10):1462-7.
