# Supplementary material for: Prevalence of neonatal hypothermia and its associated factors in East Africa: a systematic review and meta-analysis
Source: BMC Pediatr. 2020 Apr 3;20:148. doi: 10.1186/s12887-020-02024-w (PMC7118870; doi:10.1186/s12887-020-02024-w)
Supplement: Supplementary file 3 — Additional file 3: Table S3. Adjusted confounders and main findings extracted from included studies in East Africa, from January 2000–December 2019. [file 12887_2020_2024_MOESM3_ESM.docx]

**Table S3:** Adjusted confounders and main findings extracted from included studies in East Africa, from January 2000-December 2019.

| **Author** | **Adjusted confounders** | **Main findings** |
| --- | --- | --- |
| Cross-sectional studies | | |
| Byaruhanga R(1) | Parity, preterm delivery, nighttime delivery, rupture of membranes `24hrs and location of newborns in theatre | No skin to skin contact comprised 87% of hypothermic newborns .The mean birth weight was 3218g. Low birth weight newborns constituted 9/86 (10% ) among hypothermic newborns. |
| Hayelom G /(2) | - | Of the1152 live births, there were 68 deaths (63 per 1000 live births). Two thirds of deaths were attributable to prematurity 23 (34%) or asphyxia 21 (31%). In the early neonatal period, 37% were due to prematurity. |
| Abayneh G /(3) | Residence, place of delivery, gestational age, birth weight in grams, ANC follow-up, mode of delivery, body temperature, hypoglycemia, neonatal sepsis, peri-natal asphyxia, and RDS | Peri-natal asphyxia, instrumental delivery and early onset neonatal sepsis |
| Birhanu W/(4) | Age of neonate ,birth weight ,gestational age, skin to skin contact, early initiation of breast feeding, PR received ,obstetric complication during pregnancy, pregnancy type, and time of delivery | Preterm delivery, age of Neonate ≤24 h old, no skin to skin contact ,delayed initiation of breastfeeding and resuscitation at birth were significantly associated |
| Gebresilasea G/(5) | Weight of the neonate, baby breastfed within 1 hour, bathed within 24 hour, Season, obstetric complication, residence, time of delivery, number of ANC visits | Admission weight below 2500 gm., delay in initiation of breastfeeding, early bathing, admissions during cold season and presence of obstetrical complication(s) during pregnancy/labor were factors significantly associated with hypothermia. |
| Hagos T/(6) | Residence, bathing in 24 h, skin to skin contact, initiation of breastfeeding, CPR, complication, APGAR at 5 min, type of pregnancy, mode of delivery, time of born, gestational age and Wight | Delayed initiation of breastfeeding ,LBW , preterm  , low APGAR score, skin to skin contact, night time delivery , and bathed within 24 h were independent risk factors of neonatal hypothermia. |
| Wubet A /(7) | Skin to skin contact, Wearing cap, Proper wrapping, Early breast feeding, Warm transportation, Obstetric complication, Birth weight (Kg), Gestational age (weeks), and Neonatal health problem | No skin to skin contact, no wearing cap, no warm intra-facility transportation, born to mothers having obstetric complication, prematurity and neonatal health problem were significantly associated with hypothermia. |
| Mekonnen T/(8) | - | 1316 neonates were admitted in the last three years. 300 died. 93 (31%) delivered prematurely, 89 (29.7%) admitted for diagnosis of sepsis and 46 (15.3%) were those admitted due to low birth weight |
| Switchenko N/(9) | - | 55 (60%) had persistent hypothermia. 7(13 %) of the neonates who had persistent hypothermia died, 18 infants (14%) had recorded hyperthermia. Although persistent hypothermia was not associated with death (p=0.09). |
| Tewodros S/(10) | Birth weight, CPR, problem of neonate, GA, early Initiation of BF, skin-to-Skin contact, and time of delivery | Low birth weight, no skin to skin contact, night time delivery, delayed initiation of breast feeding and problems of the neonates were significantly associated with hypothermia. |
| **Cohort studies** | | |
| Alison Talbert /(11) | - | Mortality rates were higher in children with hypothermia (4/12, 33%) than those without (121/655, 18%), the timing of hypothermia did not coincide with clinical deterioration. |
| **Case control study** | | |
| Bergstrom A/(12) | - | Bathing of newborns in the first hour after delivery resulted in a significantly increased prevalence of hypothermia. |

**References**

1. Byaruhanga R, Bergstrom A, Okong P. Neonatal hypothermia in Uganda: prevalence and risk factors. Journal of tropical pediatrics. 2005;51(4):212-5.

2. Mengesha HG, Sahle BW. Cause of neonatal deaths in Northern Ethiopia: a prospective cohort study. BMC public health. 2017;17(1):62.

3. Demisse AG, Alemu F, Gizaw MA, Tigabu Z. Patterns of admission and factors associated with neonatal mortality among neonates admitted to the neonatal intensive care unit of University of Gondar Hospital, Northwest Ethiopia. Pediatric health, medicine and therapeutics. 2017;8:57.

4. Demissie BW, Abera BB, Chichiabellu TY, Astawesegn FH. Neonatal hypothermia and associated factors among neonates admitted to neonatal intensive care unit of public hospitals in Addis Ababa, Ethiopia. BMC pediatrics. 2018;18(1):263.

5. Ukke GG, Diriba K. Prevalence and factors associated with neonatal hypothermia on admission to neonatal intensive care units in Southwest Ethiopia–A cross-sectional study. PloS one. 2019;14(6):e0218020.

6. Tasew H, Gebrekristos K, Kidanu K, Mariye T, Teklay G. Determinants of hypothermia on neonates admitted to the intensive care unit of public hospitals of Central Zone, Tigray, Ethiopia 2017: unmatched case–control study. BMC research notes. 2018;11(1):576.

7. Bayih WA, Assefa N, Dheresa M, Minuye B, Demis S. Neonatal hypothermia and associated factors within six hours of delivery in eastern part of Ethiopia: a cross-sectional study. BMC pediatrics. 2019;19(1):252.

8. Mekonnen T, Tenu T, Aklilu T, Abera T. Assessment of Neonatal Death and Causes among Admitted Neonates in Neonatal Intensive Care Unit of Mizan Tepi University Teaching Hospital, Bench Maji Zone, South-West Ethiopia, 2018. Clinics Mother Child Health. 2018;15(305):2.

9. Switchenko N KE FB. Prevalence of neonatal hypothermia in a referal hospitals newborn unit in Kenya. . 2017.

10. Ebrahim TSaE. Proportion of Neonatal Hypothermia and Associated Factors among New-borns at Gondar University Teaching and Refferal Hospital, Northwest Ethiopia: A Hospital Based Cross Sectional Study.

11. Talbert A, Atkinson S, Karisa J, Ignas J, Chesaro C, Maitland K. Hypothermia in children with severe malnutrition: low prevalence on the tropical coast of Kenya. Journal of tropical pediatrics. 2009;55(6):413-6.

12. Bergström A, Byaruhanga R, Okong P. The impact of newborn bathing on the prevalence of neonatal hypothermia in Uganda: a randomized, controlled trial. Acta Paediatrica. 2005;94(10):1462-7.
